# Methyl group donors abrogate adaptive responses to dietary restriction in *C. elegans*

**DOI:** 10.1186/s12263-016-0522-4

**Published:** 2016-03-17

**Authors:** Maja Klapper, Daniel Findeis, Harald Koefeler, Frank Döring

**Affiliations:** 1 Institute of Human Nutrition and Food Science, Molecular Prevention, Christian-Albrechts University of Kiel, Heinrich-Hecht-Platz 10, 24118 Kiel, Germany; 2 Institute of Genetics, TU Braunschweig, 38106 Braunschweig, Germany; 3 ZMF—Center for Medical Research, University of Graz, Core Facility for Mass Spectrometry, Lipidomics and Metabolomics, A-8010 Graz, Austria; 4 Omics Center Graz, A-8010 Graz, Austria

**Keywords:** Dietary restriction, Lipid droplet, Choline, Methyl group donor, Thrifty phenotype, Life history traits, *C. elegans*

## Abstract

**Background:**

Almost all animals adapt to dietary restriction through alternative life history traits that affect their growth, reproduction, and survival. Economized management of fat stores is a prevalent type of such adaptations. Because one-carbon metabolism is a critical gauge of food availability, in this study, we used *Caenorhabditis elegans* to test whether the methyl group donor choline regulates adaptive responses to dietary restriction. We used a modest dietary restriction regimen that prolonged the fecund period without reducing the lifetime production of progeny, which is the best measure of fitness.

**Results:**

We found that dietary supplementation with choline abrogate the dietary restriction-induced prolongation of the reproductive period as well as the accumulation and delayed depletion of large lipid droplets and whole-fat stores and increased the survival rate in the cold. By contrast, the life span-prolonging effect of dietary restriction is not affected by choline. Moreover, we found that dietary restriction led to the enlargement of lipid droplets within embryos and enhancement of the cold tolerance of the progeny of dietarily restricted mothers. Both of these transgenerational responses to maternal dietary restriction were abrogated by exposing the parental generation to choline.

**Conclusions:**

In conclusion, supplementation with the methyl group donor choline abrogates distinct responses to dietary restriction related to reproduction, utilization of fat stored in large lipid droplets, cold tolerance, and thrifty phenotypes in *C. elegans*.

**Electronic supplementary material:**

The online version of this article (doi:10.1186/s12263-016-0522-4) contains supplementary material, which is available to authorized users.

## Background

Dietary restriction (DR) induces several adaptations, including the extension of the reproductive period, prolongation of the adult life span, and the reduction of energy turnover (Fontana and Partridge 2015). Although the life span-prolonging effect of DR and the mechanisms involved have been extensively studied in a variety of organisms, the impact of DR on the regulation of lipid droplet (LD) homeostasis is limited (Baumeier et al. 2015; Bouwman et al. 2009; Miersch and Doring 2013; Palgunow et al. 2012). LDs are evolutionarily conserved fat storage organelles containing a triacylglyceride core surrounded by a phospholipid monolayer composed mainly of phosphatidylcholine and phosphatidylethanolamine (Zhang et al. 2010b). In addition to this classical function, LDs were recently recognized to function as storage organelles for histones during embryogenesis (Cermelli et al. 2006; Li et al. 2012), assembly platforms for specific viruses (Welte 2015), and intracellular antibacterial defense systems (Anand et al. 2012). During periods of reduced food availability and/or growth, fatty acids are effectively delivered from LDs to maintain key energy-consuming processes (Barbosa et al. 2015; Lee et al. 2014; Narbonne and Roy 2009).

Functional genomic screens of the model organisms *Caenorhabditis elegans* and *Drosophila melanogaster* have been performed to identify genes that regulate LD formation and utilization (Ashrafi et al. 2003; Guo et al. 2008). These screens established that Arf1-COPI-mediated vesicular transport and the phosphatidylcholine synthesis pathway play important roles in the morphology and functionality of LDs. Many LD regulatory genes are conserved from worms to humans. Moreover, it has been shown that dietary factors, such as vaccenic acid, also regulate LD functionality (Zhang et al. 2010a). We have previously reported that in *C. elegans*, DR leads to the enlargement of LDs in the intestine and hypodermis (Miersch and Doring 2013; Palgunow et al. 2012), which are the main fat storage tissues of nematodes. However, little is known about the underlying cellular mechanism and the physiological role of this DR-induced LD phenotype. Because one-carbon metabolism regulates the homeostasis of phosphatidylcholine synthesis, lipogenesis, lipid droplet size, and lipolytic efficacy (Ehmke et al. 2014; Li et al. 2011), in this study, we used *C. elegans* to determine whether the methyl group donor choline plays a regulatory role in the enlargement of LDs and other adaptive phenotypes that are induced by DR.

## Results

### Intake of the methyl group donor choline abrogates both the enlargement of intestinal lipid droplets and the increase in the fat content of dietarily restricted worms

**Fig. 1:**
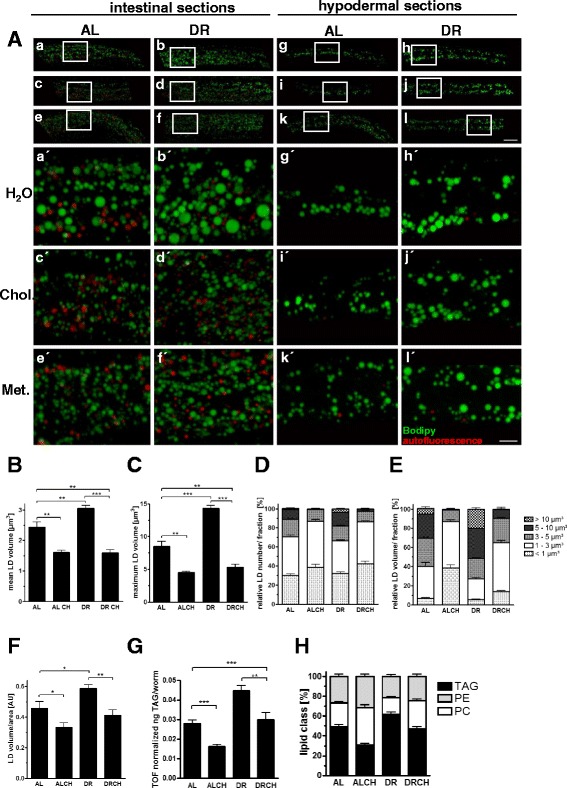
Choline and methionine supplementation abrogates the enlargement of intestinal lipid droplets and increase in fat content observed in dietarily restricted worms. **a** Lipid droplets in the mid-region of ad libitum-fed L4 larvae (*a*, *c*, *e*, *g*, *i*, *k*) and dietarily restricted L4 larvae (*b*, *d*, *f*, *h*, *j*, *l*), without (H_2_O vehicle control; *a*, *b*, *g*, *h*) or with choline (*c*, *d*, *i*, *j*) or methionine (*e*, *f*, *k*, *l*) supplementation, were visualized after short-term (20 min) vital BODIPY^™ 493/503^ staining using a ×63 objective and Zeiss Apotome equipment. Image stacks with a depth of 10 μm, consisting of 21 planes at 0.5-μm intervals, were captured and rendered as maximal projections (*a–f*). Images *a–f* show intestinal lipid droplets. In *g–l*, only the first six to eight images of the respective stacks are projected to demonstrate the lipid droplets in the upper hypodermal segment. The BODIPY^™ 493/503^ fluorescence signals (*green*) and the auto-fluorescence signals of lysosome-related organelles (*red*) are shown to study possible co-localizations of the signals. Scale bars, 20 and 10 μm (magnifications). **b**–**f** Images of intestinal lipid droplets were analyzed using the 3D spot segmentation plug-in of ImageJ software. The mean LD volume (**b**), maximum LD volume (**c**), and total LD volume are shown. The distributions of the relative number of LDs (**d**) and relative LD volume (**e**) per LD size category (<1, 1–3, 3–5, 5–10, and >10 μm^3^) are shown. **f** The summarized LD volume per area (AU, arbitrary units), a proxy indicator of the extent of fat storage, is shown (**f**). The data were derived from six to nine individual worms per feeding condition in three independent experiments. **g**, **h** The triacylglyceride (TAG), phosphatidylethanolamine (PE), and phosphatidylcholine (PC) contents of L4 larvae were assessed using thin-layer chromatography (TLC). **g** TAG content was normalized to the TOF value (time of flight), a proxy indicator of the worm’s body length. Five-microliter samples containing lipids from equal numbers of worms (166 or 333) were dotted onto each lane to ensure that the amounts of triacylglycerides and phospholipids were in the linear range of the triolein and phospholipid standard curve, respectively. The assays were conducted in duplicate, and the results of six to eight independent experiments are summarized. *AL* ad libitum, *AL CH* ad libitum condition with choline supplementation, *DR* dietary restriction, *DR CH* dietary restriction condition with choline supplementation, *TOF* time of flight, a proxy measurement of the length of the worms

Consistent with the results of our previous studies (Miersch and Doring 2013; Palgunow et al. 2012), DR was observed to cause enlargement of LDs in the intestine, which is one of the main fat storage tissues of *C. elegans* (Fig. 1a, a vs b). Because one-carbon metabolism plays a critical role in the regulation of both LD size and the extent of fat storage (Ehmke et al. 2014; Li et al. 2011), we supplemented the diet of the DR worms with the methyl group donor choline. We found that choline supplementation abrogates the enlargement of the intestinal LDs in DR worms (Fig. 1a, b vs d). Supplementation with the essential amino acid methionine, another methyl group donor, also reduced the size of the LDs in the intestine of DR worms (Fig. 1a, b vs f). Quantitative analysis revealed that the increased mean (Fig. 1b) and maximal (Fig. 1c) LD volume and the higher proportion of LDs >5 μm^3^ (Fig. 1d/e) were completely abrogated, or even overcompensated, in DR worms provided with supplemental choline. Choline supplementation also prevented the increase in the total LD volume (Fig. 1f) of DR worms. Because this LD parameter may serve as a proxy indicator of the extent of fat storage, we determined the triacylglyceride (TAG) contents of the worms. Consistent with the results of the LD analysis, the increase in the TAG content of DR worms was also abrogated by choline supplementation (Fig. 1g). The analysis of lipid classes revealed that choline supplementation increased the phosphatidylcholine content, which was reduced in worms subjected to DR (Fig. 1h). Taken together, our results showed that both the enlargement of intestinal lipid droplets and the increase in the fat content of dietarily restricted worms are abolished by dietary supplementation with the methyl group donor choline.

### Choline does not abrogate the enlargement of hypodermal lipid droplets induced by dietary restriction

**Fig. 2:**
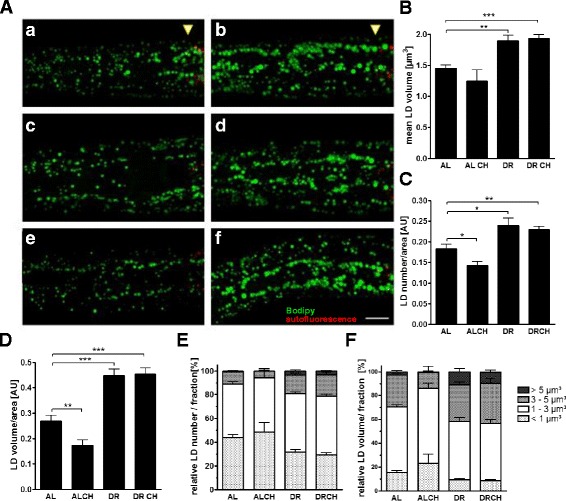
Choline and methionine supplementation do not abrogate the enlargement of the hypodermal lipid droplets of dietarily restricted worms. **a** Hypodermal lipid droplets in the heads of ad libitum-fed L4 larvae (*a*, *c*, *e*) and dietarily restricted L4 larvae (*b*, *d*, *f*) without (H_2_O vehicle control; *a*, *b*) or with choline (*c*, *d*) and methionine (*e*, *f*) supplementation were visualized using short-term vital BODIPY^™ 493/503^ staining. Image stacks with a depth of 10 μm, consisting of 21 focal planes at 0.5-μm intervals, were captured and rendered as maximum projections. *Yellow arrowheads* indicate the beginning of the intestine. The BODIPY^™ 493/503^ fluorescence signals (*green*) and the auto-fluorescence signals of lysosome-related organelles (*red*) are shown to study possible co-localizations of the signals. Scale bars, 20 and 10 μm (magnifications). **b**–**f** Quantification of hypodermal lipid droplets in the head using the 3D spot segmentation plug-in of ImageJ software. The mean LD volume (**b**), the summarized LD number/area (AU, arbitrary unit) (**c**), and the summarized LD volume/area (AU, arbitrary unit), a proxy indicator of the extent of fat storage (**D**), are shown. The distributions of the relative LD number **e** and LD volume **f** per LD size category (<1, 1–3, 3–5, >5 μm^3^) are shown. The data were derived from six to nine individual worms per feeding condition in three independent experiments. *AL* ad libitum, *AL CH* ad libitum condition with choline supplementation, *DR* dietary restriction, *DR CH* dietary restriction condition with choline supplementation

In addition to the intestine, the hypodermis functions as a major fat storage tissue in *C. elegans*. Confocal microscopy of the mid-segments (Fig. 1a, g–i) and heads (Fig. 2a, a–f) of the worms revealed that neither choline nor methionine supplementation prevented the enlargement of the hypodermal LDs in these regions of DR worms. Quantitative analyses of the hypodermal LDs localized in the head region of the worms showed that DR increased the mean LD volume (Fig. 2b), the number of LDs (Fig. 2c), the total LD volume (Fig. 2d)—a proxy indicator of the extent of fat storage—and the proportion of LDs >5 μm^3^ (Fig. 2e/f). None of these effects of DR was prevented by choline supplementation. Therefore, choline does not affect the DR-mediated enlargement of hypodermal LDs.

### Choline abrogates the delayed depletion of the fat stores of dietarily restricted worms

**Fig. 3:**
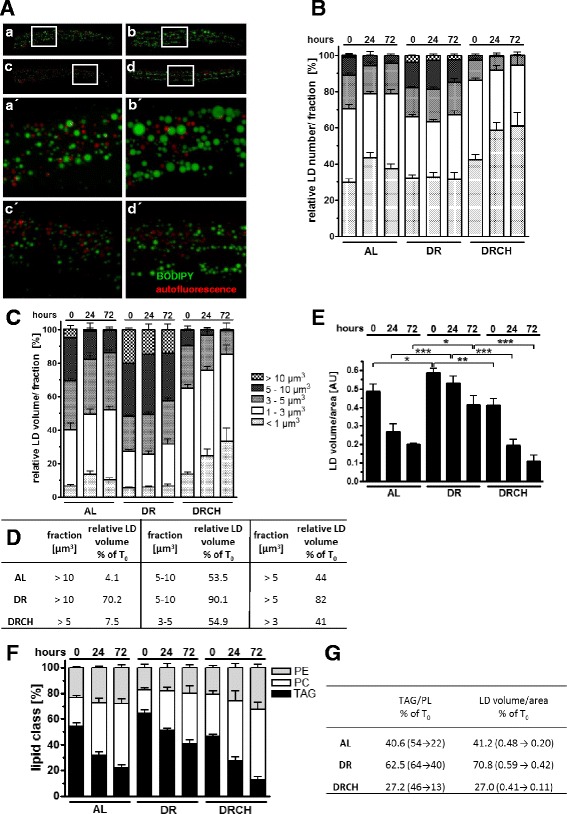
Choline abrogates the delayed depletion of the fat stores of dietarily restricted worms. **a** Depletion of lipid droplets in response to starvation (24 h) in ad libitum-fed L4 larvae (*a*, *c*) and dietarily restricted L4 larvae (*b*, *d*), without (H_2_O vehicle control; *a*, *b*), or with choline (*c*, *d*) supplementation. Lipid droplets were visualized after short-term (20 min) vital BODIPY^™ 493/503^ staining using a ×63 objective and Zeiss Apotome equipment. Image stacks with a depth of 10 μm, consisting of 21 focal planes at 0.5-μm intervals, were captured and rendered as maximum projections. The images show intestinal lipid droplets in the mid-regions of the worms. The BODIPY^™ 493/503^ fluorescence signals (*green*) and the auto-fluorescence signals of lysosome-related organelles (*red*) are shown to study possible co-localizations of the signals. Scale bars, 20 and 10 μm (magnifications). **b**–**e** Quantification of intestinal lipid droplets using the 3D spot segmentation plug-in of ImageJ software. The lipid droplets of ad libitum-fed (AL) and dietarily restricted (DR) L4 larvae were analyzed at time point T_0_ (0 h, no starvation) and in response to starvation for 24 or 72 h. The distributions of the relative lipid droplet number/fraction (**b**) and the relative lipid droplet volume/fraction (**c**) per lipid droplet size category (<1, 1–3, 3–5, 5–10, >10 μm^3^) are shown. Table **d** shows the relative lipid droplet volume in response to 72 h of starvation (% of T_0_) among different lipid droplet size categories (>10, 5–10, >5 μm^3^). **e** shows the summarized LD volume/area (AU, arbitrary unit), a proxy indicator of the extent of fat storage. The data were derived from six to nine individual worms per feeding condition in three independent experiments. **f**, **g** Triacylglyceride (TAG), phosphatidylethanolamine (PE), and phosphatidylcholine (PC) contents were assessed using thin-layer chromatography (TLC). The lipid contents of ad libitum-fed (AL) and dietarily restricted (DR) L4 larvae were analyzed at time point T_0_ (0 h, no starvation) and in response to starvation for 24 or 72 h. Table **g** shows the relative ratio of the TAG and phospholipid (PL; PE+PC) contents in response to 72 h of starvation (% of T_0_). These ratios correspond to the lipid droplet volume/area. Five-microliter samples containing lipids from equal numbers of worms (166 or 333) were dotted onto each lane to ensure that the amounts of triacylglycerides and phospholipids were in the linear range of the triolein and phospholipid standard curve, respectively. The assays were conducted in duplicate, and six to eight independent experiments are summarized. *AL* ad libitum, *DR* dietary restriction, *DR CH* dietary restriction condition supplemented with choline

Economized management of fat stores is a common adaptation to DR conditions. We therefore evaluated whether DR worms retain their fat stores more effectively than AL-fed worms (AL, ad libitum). In response to starvation (24 or 72 h), the contents of the LDs in the mid-segment of the worms were exhausted in the AL condition (Fig. 3a, a vs c), but not in the DR condition (Fig. 3a, b vs d). Quantitative analysis revealed that the large LDs (5–10 μm^3^, >10 μm^3^) of DR worms were more resistant to starvation-induced fat depletion than those of the AL-fed worms (Fig. 3b–d). Next, we evaluated the fat stores of the worms by analyzing both LD volume (Fig. 3e) and lipid content (Fig. 3f). It was consistently found using both methods of evaluation that the DR worms retained approximately 60–70 % of their initial fat stores in response to starvation, whereas the AL-fed worms retained approximately 40 % (Fig. 3e/f) of these stores. Finally, we examined whether choline supplementation would prevent the occurrence of the fat-retaining phenotype of DR worms. We found that the decrease in LD exhaustion (Fig. 3a, b vs d) and the delay in the depletion of both the large LDs >5 μm^3^ (Fig. 3b–d) and fat stores (Fig. 3e/f) in the DR worms were abolished by choline supplementation. Taken together, our results indicate that DR worms preserve fat stores more efficiently than AL-fed animals, with the larger LDs being particularly resistant to lipolysis. These adaptive responses are abolished when the diet of DR worms is supplemented with the methyl group donor choline.

### Choline abrogates the enlargement of the lipid droplets of progeny (*F*_1_) produced by dietarily restricted mothers (*P*_o_)

**Fig. 4:**
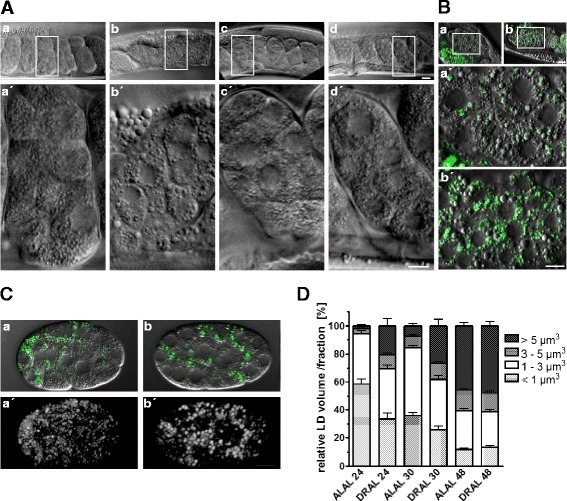
Choline and methionine abrogate the enlargement of the lipid droplets of in utero embryos (F_1_) produced by dietarily restricted mothers (P_o_)*.* **a** DIC images of in utero embryos (F_1_ generation) derived from ad libitum-fed (AL, *a*) and dietarily restricted (DR, *b*) adult worms (P_0_ generation) supplemented with choline (*c*) or methionine (*d*). Images were captured using a ×63 objective. Scale bar, 20 μm. Enlargements of the framed areas are shown in *a´–d´* (×5, scale bar, 10 μm). **b** The lipid droplet marker ATGL::GFP (*a*, *a´*) and the vitellogenin marker Vit-2::GFP (*b*, *b´*) are shown. Images were captured using a ×100 objective. Scale bar, 20 μm; ×5 magnification *a´*, *b´*, scale bar, 10 μm. **c** Lipid droplets from isolated embryos obtained from ad libitum-fed (AL, *a*) and dietarily restricted (DR, *b*) adult worms were visualized using short-term vital BODIPY^™ 493/503^ staining. Merged DIC and BODIPY^™ 493/503^ fluorescence images (*a*, *b*) and fluorescence images (*a´*, *b´*) are shown. Image stacks (*a´*, *b´*) with a depth of 10 μm, consisting of 21 focal planes at 0.5 μm intervals, were captured and rendered as maximum projections of the BODIPY^™ 493/503^ signals. **d** Quantification of intestinal lipid droplets in ad libitum-fed (AL) L1 larvae (F_1_ generation; 24 h after hatching), L1/L2 larvae (F_1_ generation; 30 h after hatching), and L4 (F_1_ generation; 48 h after hatching) obtained from ad libitum-fed (AL) and dietarily restricted (DR) adult worms (P_0_ generation). Analyses were performed using the 3D spot segmentation plug-in of ImageJ software. The distribution of the relative lipid-droplet volume/fraction ratio per lipid droplet size category (<1, 1–3, 3–5, >5 μm^3^) is shown. The data were derived from six to nine individual worms per feeding condition in three independent experiments. ALAL24/30/48, ad libitum feeding of the P_0_ generation and ad libitum feeding of F_1_ larvae 24, 30, or 48 h after hatching; DRAL24/30/48, dietary restriction of the P_0_ generation and ad libitum feeding of F_1_ larvae 24, 30, or 48 h after hatching

Because the increased LD size, higher fat content, and less depleted fat stores observed in DR worms are thrifty phenotypic traits, we speculated that maternal DR would affect LD size and fat-related phenotypes in the descendent F_1_ progeny. Differential lipid analysis revealed similar changes in both the triacylglyceride-to-phospholipid ratio and the composition of the phosphatidylcholine fatty acid species of the restricted P_0_ generation and the F_1_ embryos (F_1_ generation) of DR mothers (Additional file 1: Figure S1). These results indicate that quantitative as well as qualitative aspects of lipid metabolism are transmitted to the embryos of DR mothers. Remarkably, the F_1_ embryos showed enlarged vesicles localized throughout their bodies in utero (Fig. 4a, a vs b). These vesicles co-localized with the LD marker protein ATGL (Fig. 4b, a), but not with VIT-2, a yolk marker (Fig. 4b, b). The large vesicles of isolated embryos were BODIPY^™ 493/503^-positive (Fig. c). These results confirmed that the enlarged vesicles observed in DR-derived embryos are true LDs. A higher proportion of large LDs >5 μm^3^ were still present in DR-derived AL-fed L1/2 larvae (AL-DR, 24/30) compared with AL-derived AL-fed L1/2 larvae (AL-AL, 24/30) (Fig. 4d). This persistent effect was not observed in DR-derived L4 larvae (Fig. 4d). Importantly, enlarged LDs did not occur in the embryos (F_1_ generation) of DR mothers (P_0_ generation) that were provided a diet supplemented with either choline or methionine (Fig. 4a, b vs c, d). Taken together, the results show that maternal DR causes enlargement of the LDs of offspring and that this transgenerational effect is abolished by providing methyl group donors to the mothers.

### Choline abrogates the increased survival rates observed in the cold in dietarily restricted worms and in the larvae produced by dietarily restricted mothers

**Fig. 5:**
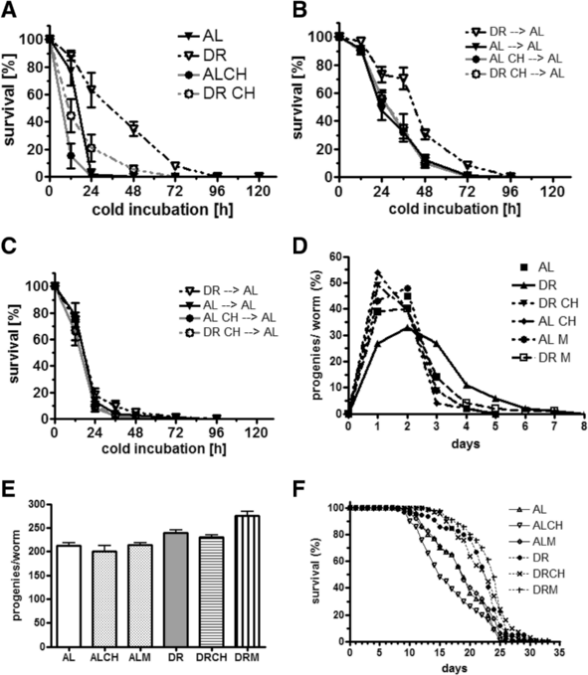
Choline abrogates the extension of the reproductive period and increased survival rate in the cold in the P_0_ and F_1_ generations, but not the increase in the life span of dietarily restricted worms. **a** Survival curves of ad libitum-fed (AL) and dietarily restricted (DR) young adult worms without (H_2_O vehicle control; AL, DR) or with choline (AL CH, DR CH) supplementation, following their transfer from 20 to 0 °C. The data were obtained in three independent experiments. **b**, **c** Survival curves of AL-fed L1 larvae (**b**) or AL-fed L4 larvae (**c**) obtained from either AL-fed or DR adult worms, without (H_2_O vehicle control; AL, DR) or with choline (AL CH, DR CH) supplementation, following their direct transfer from 20 to 0 °C. The data were obtained in three independent experiments. DR/AL→AL, DR/AL) of the P_0_ generation and AL of F_1_ larvae; DR CH →AL, DR with choline supplementation of the P_0_ generation and AL feeding of F_1_ larvae. **d**–**f** Time-course of the progeny production (**d**), length of the reproductive period (**e**), and lifetime progeny production rate (**f**) of ad libitum-fed (AL) and dietarily restricted (DR) worms, without (H_2_O vehicle control; AL, DR) or with choline (AL CH, DR CH) or methionine (AL M, DR M) supplementation. **g** Life span curves representing the typical results of two to four independent experiments using ad libitum-fed (AL) and dietarily restricted (DR) worms, without (H_2_O vehicle control; AL, DR) or with choline (AL CH, DR CH) or methionine (AL M, DR M) supplementation

In nature, DR is often coincident with lower temperatures. Thus, for the ectotherm *C. elegans* in particular, it would be favorable for DR worms to provoke adaptations that are beneficial under cold conditions. Consistent with this theory, DR worms survived the cold for longer periods than do AL-fed worms (Fig. 5a). This DR response was also abolished by choline supplementation (Fig. 5a). Similar to the persistence of the LD phenotype, DR-derived AL-fed L2 larvae (DR-AL) (Fig. 5b), but not the respective L4 larvae (Fig. 5c), were more resistant to cold than the AL-derived AL-fed L2 larvae (AL-AL). As observed for LD size, the transgenerational effect of DR on cold survival was abrogated by choline supplementation of the mothers’ diet (Fig. 5b, DRCH-AL). Thus, both DR worms and the progeny of DR mothers show higher survival rates in the cold compared with their AL-fed counterparts. These cold-adaptive responses of DR animals are abrogated by choline supplementation.

### Choline abrogates the extension of the reproductive period, but not the increase in the life span of dietarily restricted worms

The altered lipid composition, increased LD size, and higher survival rates in the cold observed in embryos derived from DR mothers might indicate that the reproduction of mothers subjected to DR is altered. As expected, DR prolonged the fecund period of the worms compared with those exposed to the AL conditions (Fig. 5d). The resulting lifetime progeny production rate, which is the best indicator of fitness, was not reduced and was even slightly increased by DR (Fig. 5e). The DR-induced extension of the reproductive period was completely abrogated by either choline or methionine supplementation (Fig. 5d). Surprisingly, the DR-mediated life span extension was only slightly reduced by choline or methionine supplementation (Fig. 5f), indicating that these methyl group donors exert specific effects on LD size, fat storage, survival in the cold, and reproduction.

## Discussion

Almost all organisms respond to reduced food intake, or DR, via alternative life history traits that ensure survival, growth, and reproduction. In the present study, we applied a standardized DR regime (Miersch and Doring 2013; Palgunow et al. 2012) during postembryonic development. As this DR regime prevents developmental delay and growth retardation, it mimics a moderate, but sufficient food intake. The phenotypes resulting from implementing this DR regime include the accumulation of fat in large LDs, delayed depletion of fat stores, and prolongation of the fecund period without a reduction of the lifetime progeny production rate. Thus, the observed DR-induced phenotypes are highly consistent with the life history theory. Another basic theory argues that a food-restricted mother modifies the phenotype of her progeny such that they are prepared for survival in a poor nutritional environment (Barker 2001). The enlargement of the LDs and the accumulation of triacylglycerides in embryos derived from DR mothers might represent such a thrifty phenotype, assuming that the accumulated, larger embryonic LDs are more resistant to starvation-induced depletion, as observed in the mothers.

As mentioned above, our findings regarding the DR-induced phenotypes are in good agreement with the life history theory and the thrifty phenotype theory. These types of ultimate explanations are insufficient to explain the biological functions of the phenotypes in terms of immediate physiological or environmental factors. In searching for such factors, we consider that animals with a genetic deficiency in methyl group donors phenocopies the observed DR-induced effects. For example, *C. elegans* mutants deficient in the synthesis of SAM, the main methyl group donor found in most organisms, show enlarged LDs during both the adult and embryonic stages, increased fat stores, reduced levels of phosphatidylcholine, and delayed depletion of fat stores (Ehmke et al. 2014; Hansen et al. 2005; Walker et al. 2011). Several animal- and patient-based studies have suggested that a diet deficient in methyl group donors is an important factor in the etiology of non-alcoholic fat liver (Leermakers et al. 2015). For example, a choline-deficient diet causes hepatic fat accumulation (Rinella and Green 2004; Zeisel et al. 1991). We suggest that endogenous and exogenous methyl group donors are critical under DR conditions and that the resulting activity of one-carbon metabolism in turn regulates LD size and fat storage. This phenomenon is particularly true for *C. elegans* and other nematodes because their food source, *Escerichia coli*, does not contain phosphatidylcholine. Accordingly, we found that dietary supplementation with choline as well as methionine abrogates the DR-induced fat-related phenotypes. Interestingly, folic acid and vitamin B_12_ are also involved in determining the activity of one-carbon metabolism. Restriction of these vitamins may be related to the fat-related phenotypes observed under DR. A study in rats has shown that vitamin B_12_ deficiency causes increased fatty acid synthesis (Matlib et al. 1979)*.* All of these findings support the view that one-carbon metabolism is a critical gauge of food availability and a key pathway involved in regulating the adaptive storage and utilization of fat.

The most abundant phospholipid in the LD monolayer is PC, which can be synthesized from choline (via the Kennedy pathway), or via the SAM-dependent methylation of phosphoethanolamine (in nematodes and plants) or phosphatidylethanolamine (in mammals) (Brendza et al. 2007; Palavalli et al. 2006). It has been shown that depletion of PC or loss-of-function mutations of the genes that mediate methylation-dependent PC synthesis (*sams-1* and *pmt-1*) leads to enlargement of LDs (Bartz et al. 2007; Guo et al. 2008; Szymanski et al. 2007; Walker et al. 2011). Further studies revealed that LD enlargement occurs when there is a reduced level of PC due to fusion of the existing LDs (Guo et al. 2008; Krahmer et al. 2011; Li et al. 2011; Walker et al. 2011). Based on these findings, we propose that the expansion of LDs induced by DR is caused by the decreased availability of methyl group donors and subsequent reduction of PC levels, which in turn promotes LD fusion. This hypothesis is supported by the observation that dietary supplementation with the methyl group donors choline and methionine increases the PC content and decreases LD size in DR worms.

Remarkably, embryos derived from DR mothers also exhibit a decreased PC content and very large LDs. Both of these phenotypes can be abolished by choline or methionine supplementation of the restricted diet of the mothers, indicating that these methyl group donors are provided to the embryos maternally. Consistent with this conclusion, the embryos of a methyl group donor-deficient mutant (*sams-1*) also exhibit large LDs. As enlarged embryonic LDs as well as cold tolerance are also observed in DR mother-derived L1/2 larvae, we suggest that the maternally provided methyl group donors persist in the progeny. Thus, the existence of a relationship between the availability of methyl group donors, PC contents, and LD size provides an explanation for the observed DR-induced fat-related phenotypes and their abolishment through choline or methionine supplementation. As the reduced PC content, increased LD size, and adaptation to cold are inherited by the next generation and can be abolished by supplementation with methyl group donors, the one-carbon metabolism of the mothers may play an important role in the occurrence of these thrifty phenotypes. Nevertheless, we cannot exclude that the inherited effect has something to do with histone methylation and subsequent gene expression. This should be studied in future experiments focusing on epigenetic effects.

We found that the starvation-induced depletion of fat stores, particularly that of the large LDs, is reduced in DR worms. This fat-sparing effect can also be abolished by supplementation with methyl group donors. In *Drosophila*, it has been shown that DR increases starvation resistance (Burger et al. 2007). Because the surface-to-volume ratio is lower in larger LDs than in smaller LDs, the rate of degradation of the former by lipases might be decreased. Consistent with this hypothesis, a *C. elegans* mutant (*dhs-28*) deficient in peroxisomal β-oxidation is characterized by extremely large LDs that are more resistant to lipase-mediated lipolysis compared with smaller LDs (Zhang et al. 2010a). Moreover, studies in flies have shown that the large LDs that form due to reduced PC levels are more resistant to lipolysis (Guo et al. 2008; Krahmer et al. 2011). In mutant mice, the generation of small LDs leads to enhanced lipolysis (Nishino et al. 2008). The size of LDs is functionally important in the supply of fatty acids. From this point of view, a decreased rate of supply of fatty acids derived from large LDs may contribute to the prolonged reproductive period of DR worms. This hypothesis should be tested in future studies using mutants that are not responsive to starvation-induced fat depletion.

## Conclusions

We have demonstrated that subjecting *C. elegans* to a moderate DR regime during the postembryonic period induces the prolongation of the reproductive period, the accumulation and delayed depletion of large LDs and whole-fat stores, an increased survival rate in the cold, the enlargement of LDs, and a higher level of cold resistance in the filial generation. Because all of these effects are prevented by dietary supplementation with methyl group donors, we conclude that one-carbon metabolism is a critical gauge of food availability, a key pathway in regulating the adaptive storage and utilization of fat, and an important determinant for the induction of thrifty phenotypes.

## Methods

### Worm strains, culture, dietary restriction

The wild-type strain used in this study was Bristol N2. A VS20 strain expressing an ATGL-1::GFP fusion protein [atgl-1p::atgl-1::GFP] was used as a lipid droplet reporter strain. The strain pwls[vit-2p::vit-2::GFP] was used for the visualization of vitellogenin-containing yolk granules. The worms were cultured at 20 °C on nematode growth medium (NGM) plates using *E. coli* OP50 as the food source, following standard methods (Brenner 1974). For the supplementation experiments, solutions of choline (10 mM), ethanolamine (10 mM), betaine (10 mM), or methionine (1 mM) were spread on the bacterial lawn. Age synchronization of the nematodes was achieved through hypochlorite treatment of gravid adults. Dietary restriction of the worms was achieved as previously described (Palgunow et al. 2012). In short, adjusted optical densities (250 μl, OD_600_ 0.7 or 1.5) of the *E. coli* strain OP50 were spread onto antibiotic-free agar plates (without peptone) and incubated for 16 h at 37 °C. This leads to different amounts of bacteria per agar plate depending on the OD of seeded OP50. To further standardize food availability per worm, 500 synchronized embryos were sorted onto AL and dDR agar plates by flow cytometry and were cultivated at 20 °C until reaching the L4 or adult stage. To exclude starvation, L4 larvae and adult worms were transferred daily to fresh agar plates.

### Number of progeny and reproductive period

To determine the number of progeny, 48 h after synchronization and cultivation under a particular condition, L4 larvae were placed on plates containing a ring of palmitic acid to prevent their escape. Five worms per plate were cultivated for an additional 48 h. This time point was considered day 1 of adulthood and progeny production. The animals were subsequently transferred to new plates at 24-h intervals until the end of their reproductive period. The progeny on the residual plates were counted the day after the adults were transferred. Three plates were analyzed for each condition in three independent experiments. The reproductive period was calculated as the period during which 95 % of the progeny were produced.

### Survival rate under cold conditions

Immediately before the worms were shifted to 0 °C, the NGM plates were divided into four segments that were placed in different positions in the incubator. The nematodes were transferred to 0 °C as larvae or young adults, and survival was monitored at specific time points. The samples were analyzed in triplicate for each condition and time point. The survival rate was calculated as the percentage of dead individuals after 24 h of recovery at 20 °C among the total number of worms that had been counted immediately after incubation at 0 °C. Worms were scored as dead if they did not respond when gently touched with a platinum wire.

### Starvation experiments

Synchronized worms were grown for 48 h on NGM agar plates until reaching the L4 stage. Approximately 4000 worms were harvested from 10 to 12 NGM plates/condition and were washed three times with M9 buffer. Control worms (0 h of starvation) with or without choline supplementation were immediately sorted using a COPAS Biosort flow cytometric system into Precellys vials (1000 worms/sample) and were stored at −80 °C until further processing. To induce starvation, worms were cultivated in 15-ml Falcon tubes in a Nutator mixer in 3 ml of M9 buffer supplemented with an antibiotic (dilution: 1:100, Cell Culture Guard, AppliChem) and choline or the vehicle (H_2_O). After 24 and 72 h of starvation, the worms were collected as described above for the control worms (0 h of starvation). The samples were used for TLC and protein quantification. An aliquot of live worms grown under each condition (0, 24, or 72 h of starvation) was subjected to vital BODIPY^™ 493/503^ staining for analysis of the number and size of the lipid droplets.

### BODIPY^™ 493/503^ vital staining and fluorescence imaging

To visualize fat storage in worms and embryos, vital BODIPY^™ 493/503^ (Invitrogen, Darmstadt, Germany) staining was performed as previously described (Klapper et al. 2011). Briefly, freshly harvested worms were washed three times with M9 buffer and were incubated in 500 μL of BODIPY^™ 493/503^ solution (6.7 μg/μl in M9 buffer) for 20 min. After washing twice with M9, the worms were anesthetized using sodium azide (1 %) and immediately examined via microscopy. BODIPY^™ 493/503^ fluorescence was visualized using an Axio Imager system and a Z1 microscope equipped with a 38 HE filter (excitation: BP 470/40, beam splitter: FT 495, emission: BP 525/50), coupled to an apotome-sectioning system (ApoTome.2, Zeiss). To distinguish between BODIPY^™ 493/503^-positive structures and auto-fluorescent lysosome-related organelles, auto-fluorescence was imaged using the DAPI channel. GFP fluorescence was visualized with a 38 HE filter (excitation BP 470/40; beam splitter FT 495; emission BP 525/50).

### Analysis of the number and size of lipid droplets

BODIPY^™^ 493/503***-***stained lipid droplets localized in selected regions were imaged via 3D fluorescence microscopy using an Axio Imager Z1 microscope and a Plan-Apochromat ×63/1.3 oil immersion objective. The apotome-sectioning system was used to collect z-stacks with a step size of 0.5 μm. The z-stacks comprised 21 planar images (format, 138.4 × 104 μm; 692 × 520 pixels). Images were captured using an AxioCam MRm (Zeiss) system. ImageJ software (version 1.47 h) and the 3D spot segmentation plug-in were used to analyze the z-stacks. BODIPY^™ 493/503^-positive structures were automatically identified, and the volume of each droplet was evaluated by totaling the number of voxels per droplet. If necessary, the brightness and contrast levels were manually adjusted before performing the calculations.

### Thin-layer chromatography

After thawing worms stored at −80 °C, worms collected using the COPAS Biosort system were analyzed by thin-layer chromatography (Matyash et al. 2008). Lipids were extracted from 1000 or 2000 homogenized worms (*Precellys24* homogenizer; full speed; 3 × 10 s) using a solution containing 375 μl of methanol, 1250 μl of methyl-tert-butyl-ether [63], and 312 μl of H2O (3:10:2.5 [v]) and were dissolved in 30 μl of chloroform. Probes were applied to *Polygram SIL G* pre-coated TLC sheets (20 × 20 cm; 0.2-mm silica gel; Macherey-Nagel) using hexane/diethyl-ether/formic acid (20:20:1 [v]) as the mobile phase for TAG and phospholipids. A 5-μl aliquot of worm extract was applied to each lane to ensure that the amounts of TAGs and phospholipids were within the linear range of the standard curves. The assays were conducted in duplicate. The lipid spots were stained for 20 s in a dip solution containing 10 % copper (II) sulfate, 8 % phosphor acid, and 5 % methanol. Quantification was achieved by calculating the spot intensity per area (pixel/μm^2^) using *AlphaEaseFC* (BioRad) software. Triolein (Sigma Aldrich, Germany) and a phospholipid mixture (Sigma Aldrich, P3817) were used as standards. All of the results are presented as the mean values for the standard and diluted probes.

### Lipid composition analysis

Embryos were washed three times with M9 buffer supplemented with Tween 20 (1:100,000) to prevent the embryos from attaching to the tubes. Defined numbers of embryos that were sorted using the COPAS Biosort system were then transferred to chloroform-rinsed Pyrex tubes and were immediately frozen at −80 °C until being utilized for mass spectroscopy. The contents of the fatty acids triacylglyceride, phosphatidylcholine, and plasmalogen were analyzed using a high-resolution LC-MS/MS platform (Fauland et al. 2011) equipped with a Lipid Data Analyzer (Hartler et al. 2011). The results were normalized according to the contents of different fatty acid classes, the sample weight, and the protein content.

### Statistical analysis

Statistical analysis was performed using Microsoft Excel (2003) and GraphPad Prism (Version 4.0) software. Unless otherwise noted, the data represent the mean ± SEM of at least three independent experiments. The significance of the differences was calculated using an unpaired two-tailed *t* test. Welch’s correction was applied if the variances were different. Differences were considered significant at *p* < 0.05 (*), *p* < 0.01 (**), and *p* < 0.001 (***).

## Additional file

Additional file 1: Figure S1. Similar changes of the triglyceride-to-phospholipid ratio and the fatty acid composition of phosphatidylcholine (PC) in dietary restricted L4 larvae (P0 generation) compared to embryos (F1 generation) obtained from dietary restricted worms. (PPTX 106 kb)
