# Supplementary material for: Methyl group donors abrogate adaptive responses to dietary restriction in C. elegans
Source: Genes Nutr. 2016 Mar 17;11:4. doi: 10.1186/s12263-016-0522-4 (PMC4959552; doi:10.1186/s12263-016-0522-4)
Supplement: Additional file 1: Figure S1. — Similar changes of the triglyceride-to-phospholipid ratio and the fatty acid composition of phosphatidylcholine (PC) in dietary restricted L4 larvae (P0 generation) compared to embryos (F1 generation) obtained from dietary restricted worms. (PPTX 106 kb) [file 12263_2016_522_MOESM1_ESM.pptx]

## Slide 1
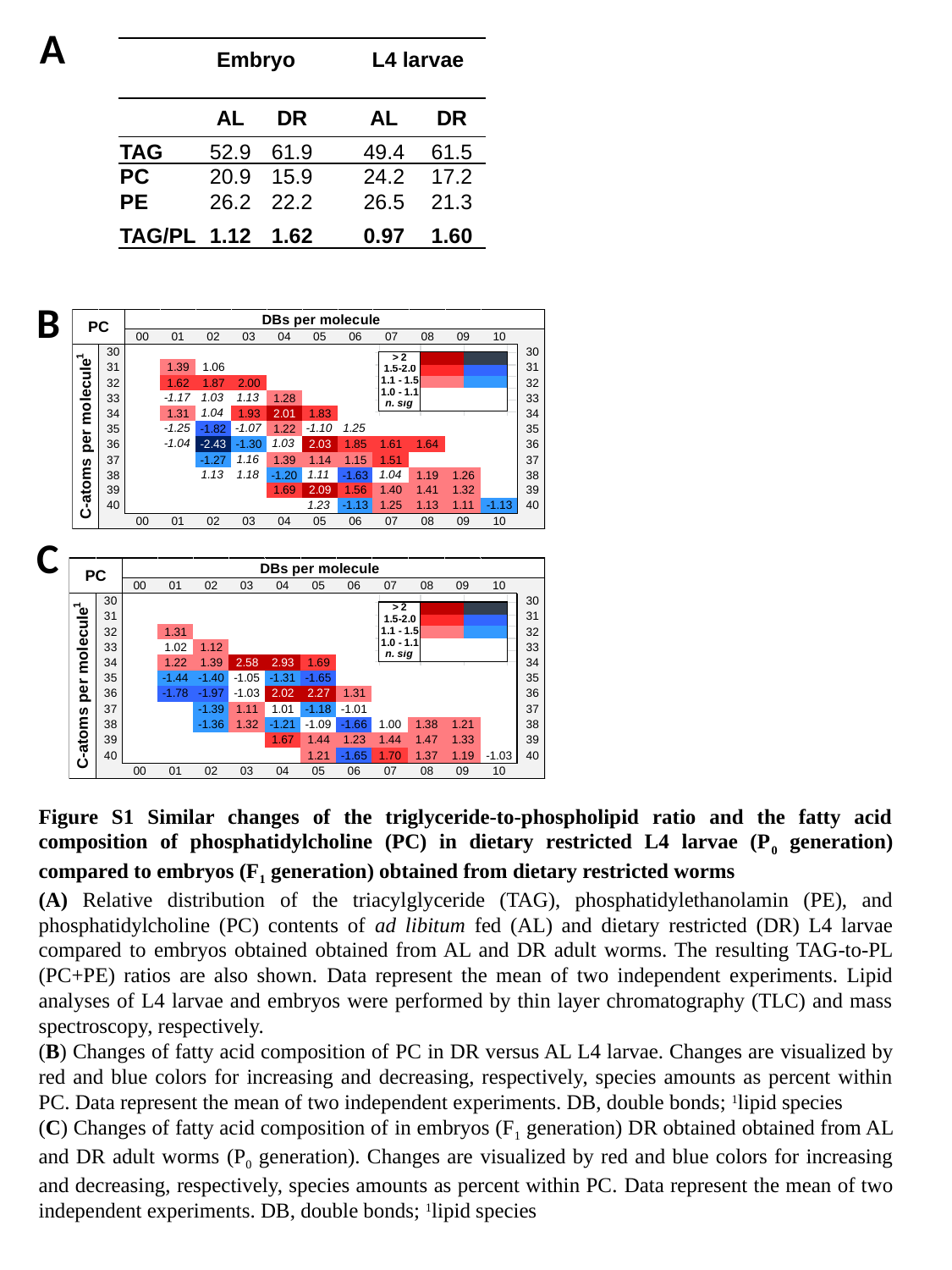

A
| Embryo L4 larvae | | | | | |
| --- | --- | --- | --- | --- | --- |
| | AL | DR | | AL | DR |
| TAG | 52.9 | 61.9 | | 49.4 | 61.5 |
| PC | 20.9 | 15.9 | | 24.2 | 17.2 |
| PE | 26.2 | 22.2 | | 26.5 | 21.3 |
| TAG/PL | 1.12 | 1.62 | | 0.97 | 1.60 |
B
C
Figure S1 Similar changes of the triglyceride-to-phospholipid ratio and the fatty acid composition of phosphatidylcholine (PC) in dietary restricted L4 larvae (P0 generation) compared to embryos (F1 generation) obtained from dietary restricted worms
(A) Relative distribution of the triacylglyceride (TAG), phosphatidylethanolamin (PE), and phosphatidylcholine (PC) contents of ad libitum fed (AL) and dietary restricted (DR) L4 larvae compared to embryos obtained obtained from AL and DR adult worms. The resulting TAG-to-PL (PC+PE) ratios are also shown. Data represent the mean of two independent experiments. Lipid analyses of L4 larvae and embryos were performed by thin layer chromatography (TLC) and mass spectroscopy, respectively.
(B) Changes of fatty acid composition of PC in DR versus AL L4 larvae. Changes are visualized by red and blue colors for increasing and decreasing, respectively, species amounts as percent within PC. Data represent the mean of two independent experiments. DB, double bonds; 1lipid species
(C) Changes of fatty acid composition of in embryos (F1 generation) DR obtained obtained from AL and DR adult worms (P0 generation). Changes are visualized by red and blue colors for increasing and decreasing, respectively, species amounts as percent within PC. Data represent the mean of two independent experiments. DB, double bonds; 1lipid species
